# EASL–EASD–EASO Clinical Practice Guidelines for the management of non-alcoholic fatty liver disease: guidelines, clinical reality and health economic aspects

**DOI:** 10.1007/s00125-016-3941-4

**Published:** 2016-04-07

**Authors:** Hermann Toplak, Rudolf Stauber, Harald Sourij

**Affiliations:** Lipid Clinic, Department of Medicine, Medical University of Graz, Auenbruggerplatz 15, A 8036 Graz, Austria; Department of Medicine, Division of Gastroenterology and Hepatology, Medical University of Graz, Graz, Austria; Department of Medicine, Division of Endocrinology and Diabetology, Medical University of Graz, Graz, Austria

**Keywords:** Differential diagnosis of steatosis, Hepatic steatosis, Lifestyle intervention, Liver biopsy, Milan Declaration 2015, Obesity-related diseases, Population strategies

An increase in the prevalence of obesity and the metabolic syndrome as a result of changes in eating habits, both quantitative (over-eating) and qualitative (e.g. high fructose consumption), and a lack of exercise contribute to fatty liver disease. Non-alcoholic fatty liver disease (NAFLD) is a clinical histological spectrum ranging from steatosis to non-alcoholic steatohepatitis (NASH) and affects more than 30% of the general population [1].

The European Association for the Study of the Liver (EASL), the European Association for the Study of Diabetes (EASD) and the European Association for the Study of Obesity (EASO) have recently published the first comprehensive joint evidence-based Clinical Practice Guidelines for the management of NAFLD [2].

While reading these important guidelines two critical questions emerge: (1) is this an algorithm that is likely to be followed in routine care, and (2) what are the health economic implications and therefore what is the likelihood of these guidelines being implemented by healthcare systems?

While previous guidelines have suggested similar algorithms (for examples see [3, 4]), a recently published survey by Rinella et al indicates that only a minority of gastroenterologists follow them. Less than 25% of the participating specialists have performed liver biopsies for the diagnosis for NASH when indicated [5], leaving NASH under-diagnosed even in gastroenterology and hepatology clinics. Thus, while the guidelines are clear and evidence based, the clinical reality remains sobering.

If the resources needed are weighed up against the clinical relevance of the findings in terms of treatment decision making, it is questionable whether liver biopsies should be performed on a large scale to differentiate steatosis from NASH. As long as the major treatment option for both conditions is lifestyle intervention including diet and exercise, with limited evidence for pharmacological treatment, discussions about the indications for biopsy-based differential diagnosis, risk of the intervention and the possible financial implications will and have to continue. Despite the fact that no drug has been approved for the indication of NASH, and guidelines recommend that pharmacological treatment should be reserved for those with biopsy-proven NASH, particularly those with significant fibrosis, in reality, treatment initiation is often solely based on clinical judgement without biopsy [5]. This observation again highlights the urgent need for reliable non-invasive biomarkers and imaging tools to differentiate NASH from steatosis, which would allow broader acceptance and more targeted treatment.

However, even if evidence-based and approved pharmacological treatment was available, it is questionable to what extent local health budgets may be able to offer it to the individual patient. This raises the additional importance of population strategies. Obesity has been developing to a central disease for many diseases, a gateway for ill health [6]. Given that the obesity epidemic is undoubtedly the strongest contributor to the burden of fatty liver disease in the 21st century, the EASO has announced the Milan Declaration 2015 [7]. This calls for action on obesity, which has emerged as the most prominent single disease of our time, hepatic steatosis being one of the consequences in many patients. Societal changes are called upon which might help the individual patient, too, such that the gap between what we can afford on an individual level and the needs of the population as a whole could be bridged. We think such population strategies should be mandatory everywhere.

In the meantime the present Clinical Practice Guidelines provide the best available evidence and should be followed to provide the best possible treatment for our patients. However, assessment of how the guidelines are translated into clinical practice in Europe and what the particular limitations are, is critical in order to address these issues in future updates.

## Abbreviations

EASO: European Association for the Study of Obesity
NAFLD: Non-alcoholic fatty liver disease
NASH: Non-alcoholic steatohepatitis
